# Comparison of the root canal debridement ability of two single file systems with a conventional multiple rotary system in long oval-shaped root canals: In vitro study

**DOI:** 10.4317/jced.52977

**Published:** 2017-08-01

**Authors:** Elham khoshbin, Abbas Shokri, Zakieh Donyavi, Shahriar Shahriari, Golsa Salehimehr, Maryam Farhadian, Zeinab Kavandi

**Affiliations:** 1 Assistant Professor, Department of Endodontics, Dental School, Hamadan University of Medical Sciences, Hamadan, Iran; 2 Assistant Professor, Department of Oral and Maxillofacial Radiology, Dental School, Dental Research Center, Hamadan University of Medical Sciences, Hamadan, Iran; 3 Assistant Professor, Department of Endodontics, Dental School, Hamadan University of Medical Sciences, Hamadan, Iran; 4 Assistant Professor, Department of Endodontics, Dental School, Kashan University of Medical Sciences, Kashan, Iran; 5 Assistant Professor, Modeling of Noncommunicable Disease Research Center, Department of Biostatistics, Hamadan University of Medical Sciences, Hamadan, Iran; 6 Postgraduate Student of Endodontics, Department of Endodontics, Dental School, Hamadan University of Medical Sciences, Hamadan, Iran

## Abstract

**Background:**

This study sought to compare the root canal debridement ability of Neolix, Reciproc and ProTaper rotary systems in long oval-shaped root canals.

**Material and Methods:**

Eighty five extracted single-rooted human teeth with long oval-shaped single root canals were selected and divided into three experimental groups(n=25) and one control group (n= 10). Root canals were filled with Vitapex radiopaque contrast medium and prepared with Neolix, Reciproc or ProTaper systems. The control group only received irrigation. Digital radiographs were obtained at baseline and postoperatively and subjected to digital subtraction. The percentage of reduction in contrast medium was quantified at 0-5 mm and 5-10 mm distances from the apex. The data were analyzed using one-way ANOVA and t-test.

**Results:**

The mean percentage of the contrast medium removed was not significantly different in the 0-5mm segment among the three groups (*P*=0.6). In the 5-10mm segment a significant difference was found in this regard among the ProTaper and Reciproc groups (*P*=0.02) and the highest mean percentage of contrast medium was removed by ProTaper. But, difference between ProTaper and Neolix as well as Neolix and Reciproc was not significant. In Neolix (*P*=0.024) and Reciproc (*P*=0.002) systems, the mean percentage of the contrast medium removed from the 0-5mm segment was significantly greater than that in 5-10mm segment; however, this difference was not significant in ProTaper group (*P*=0.069).

**Conclusions:**

Neolix single-file system may be a suitable alternative to ProTaper multiple-file system in debridement of long oval shaped canals.

** Key words:**Root Canal Preparation, Debridement, Root Canal Therapy.

## Introduction

Mechanical preparation of the root canal system (RCS) is a major step in achieving a successful endodontic treatment. Despite the technical advances in endodontics, root canal preparation is still highly influenced by the complex and variable anatomy of the RCS (1,2). Studies have shown that despite different cleaning and shaping techniques and new instruments for more efficient preparation of root canals, some areas of the RCS still remain untouched (1,3). This is particularly important in oval-shaped root canals (4).

Root canals with maximum root canal diameter twice the minimum root canal diameter are referred to as oval-shaped canals. Oval-shaped root canals with maximum root canal diameter 2-4 times the minimum root canal diameter are referred to as long oval-shaped canals (5). Due to complicated anatomy of these canals, 30 to 40% of the root canal walls especially the buccal and lingual walls remain unprepared during the process of root canal preparation (4).

Introduction of nickel titanium (Ni-Ti) rotary instruments significantly enhanced mechanical preparation of the RCS (6).

Considering the gap of information on the quality and efficacy of the newly introduced endodontic systems especially in oval-shaped root canals, this study aimed to compare the debridement efficacy of Neolix (Neolix, Paris, France), Recirpoc (VDW, Munich, Germany) and conventional ProTaper (Dentsply Maillefer, Ballaigues, Switzerland) for preparation of long oval-shaped root canals.

## Material and Methods

This *in vitro*, experimental study was conducted on 85 single-rooted single-canal human teeth with long-oval shaped root canals. The teeth had been extracted for orthodontic reasons and periodontal problems. The research protocol was approved by the institutional Ethics Committee of the Vice Chancellor of Research, Hamadan University of Medical Sciences(Protocol No:IR.UMSHA.REC.1394.97). The teeth were randomly divided into three groups of 25 and one control group of 10. The inclusion criteria were as follows: (I) Freshly extracted teeth with a single long oval-shaped root canal (the buccolingual to mesiodistal ratio of 2.5 or higher at 5mm distance from the apex) (II) Closed apex and (III) No cracks, curvature, anomaly, fracture, extensive caries, root resorption or previous endodontic treatment.

Access cavity was prepared using a #2 round bur( Brassler, Savannah, Ga) and high speed hand piece; LA Axxess kit (Sybron Endo, Orange, CA, USA) was then used to standardize the access cavity walls; next, #2, 3 and 4 Gates Glidden drills (Dentsply Maillefer, Ballaigues, Switzerland) with low-speed hand piece were used for flaring of the coronal section of the root canal to 2-3mm below the cementoenamel junction (CEJ). Root canal was rinsed with 5mL of 5.25% sodium hypochlorite solution (Chi-min, Tehran, Iran) using a 27 gage needle. A #10 K-file (Mani Inc., Tokyo, Japan) was used to maintain root canal patency. The file was introduced into the canal until the file tip was seen at the apex. Of this length, 0.5mm was subtracted to obtain the wor-king length. Root canals were then filed with #15 and #20 K-files to obtain a glide path; 3mL of 5.25% sodium hypochlorite solution was used for rinsing of the root canal between filings. Final rinse was done with 3mL of sterile saline (Daroupakhsh, Tehran, Iran) and the root canals were dried with paper points (Ariadent, Tehran, Iran). Next, the entire root canal space was filled with radiopaque contrast medium (Vitapex Nep Dental International Inc., WA, USA) using Vitapex microtip to assess the efficacy of root canal debridement (7). A #15 K-file was used to enhance distribution of medium along the working length. Radiographs were taken again to ensure the entire root canal was filled with radiopaque medium and there was no void.

**Figure 1:**
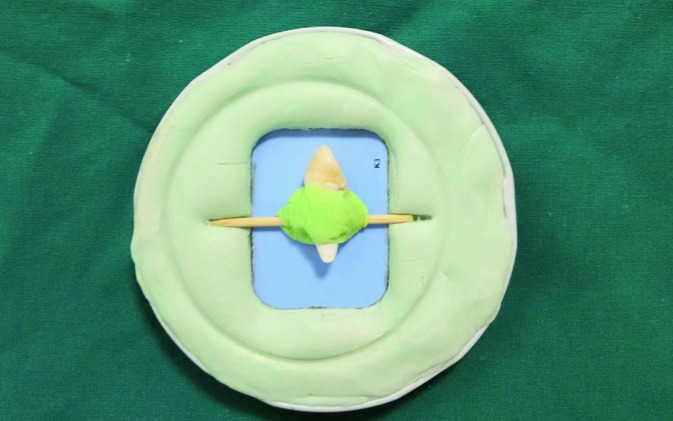
Tooth and film mounted in putty.

To perform digital subtraction radiography, we had to standardize and match the exposure settings including the angle of exposure, radiation dose, type of sensor and location of tooth on the sensor before and after the experiments. To ensure the same location of teeth on the sensor, first a toothpick was fixed to the proximal surface of the tooth using acrylic resin (Acropars, Tehran, Iran). Then, an impression was taken from the position of the sensor, position of the tube and the two ends of the toothpick using putty impression material (Fig. 1). All the teeth were radiographed as such (Minray, Soredex, Tuusula, Finland) with the exposure settings of 60 kVp, 6mA and 0.12s time using PSP sensor (Optime, Soredex, Tuusula, Finland). This radiograph was considered as the baseline radiograph. Instrumentation was then performed in Reciproc, Neolixand ProTaper groups according to the manufacturers’ instructions by the same operator as follows:

Root canal preparation with ProTaper rotary system(P):

In the P group, root canals were prepared using ProTaper files. This system includes three files for shaping and three files for finishing. Shaping files include SX, S1 and S2 with ISO 19, 17 and 20 tip diameters. The finishing files include F1 and F2 with ISO 20 and 25 tip diameters. ProTaper files were used in the P group in the following order: S1 until resistance is met, SX until resistance is met, S1 to the working length, S2 to the working length, F1 to the working length and F2 to the working length. The files were used at 300 rpm speed and 5 N/Cm torque. An Endo IT electric motor (VDW, Munich, Germany) was used to control the speed and torque. Each file was used for flaring of four teeth.

Root canal preparation with Reciproc rotary system(R):

In the R group, a #20 K-file was first introduced to the root canal. Then, #25 Reciproc was activated in a reciprocating motion by VDW silver electric motor (VBW GmbH, Munich, Germany) and was gradually introduced to the canal with pecking motion with 3mm range and brushing motion. After three pecking motions, the instrument was removed and the root canal was rinsed with 3mL of saline. This procedure was repeated three times. A #10 K-file was repeatedly used to ensure root canal patency to the working length (8).

Root canal preparation with Neolix single-file rotary system(N):

In the N group, Endo IT electric motor was used to control the speed and torque; the speed was adjusted at 300-500 rpm and 1.5 N/Cm torque with pecking and brushing motions. First, C1 file was used to flare the coronal third and eliminate dentin barriers with brushing motion only in the coronal area. Next, A1 file was passively used for preparation of the middle and apical thirds. During filing and after three to four brushing motions, the root canals were rinsed with saline and patency was ensured using #15 K-file. Eventually, the file with pecking motion was used to the working length to complete shaping of root canal.

**Figure 2:**
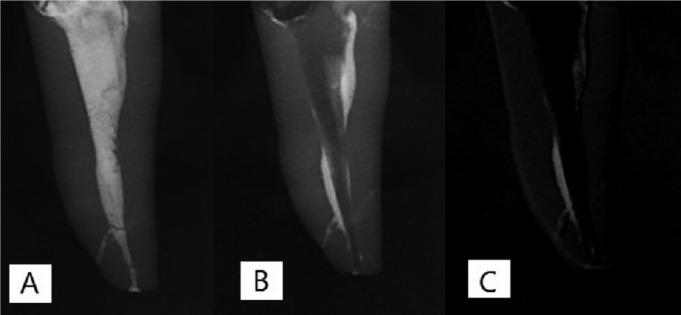
A) Tooth prior to root canal preparation B) Tooth after root canal preparation C) Digital subtraction radiograph.

Time of instrumentation in all groups was averagely four minutes. During filing, the root canals were filled with saline. The volume of irrigating solution was 20mL for each tooth. To prevent washout of radiopaque medium, sodium hypochlorite was not used. In the control group, root canals were rinsed with 20mL of saline but filing was not done (7). Pre- and post-instrumentation digital radiographs of the teeth were superimposed to determine the root canal debridement ability. The percentage of reduction of contrast medium was quantified and considered as the criterion for the ability of instrument for root canal debridement. This percentage was calculated using digital subtraction radiography(Image J; National Institutes of Health,Bethesda,MD) (Fig. 2). The root canal debridement ability of the three systems was evaluated in 0-5 and 5-10mm segments (distance from the apex).

-Statistical analysis:

Data were analyzed using SPSS version 19 (Microsoft, IL, USA) and descriptive statistics. The Kolmogorov Smirnov test, one-way ANOVA, t-test and Tukey’s test were used for data analyses. *P*<0.05 was considered statistically significant.

## Results

The mean values were compared using one-way ANOVA and t-test.  Table 1 presents the mean values of the percentage of contrast medium removed from the 0-5mm segment. The results showed that the mean percentage of the contrast medium removed was not significantly different in the 0-5mm segment among the three groups (*P*=0.6).

**Table 1:**
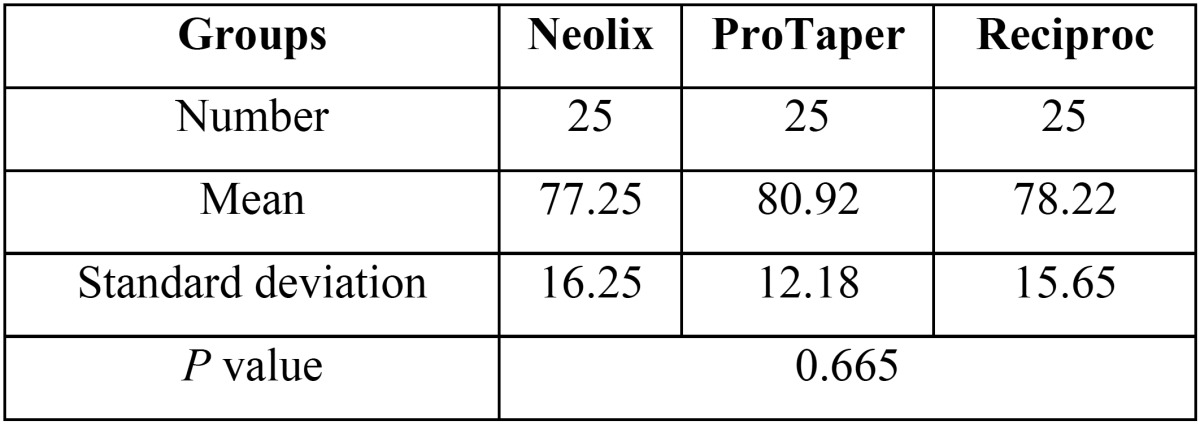
Descriptive statistics of the percentage of medium removed from the root canals in the three groups in 0-5mm segment.

 Table 2 presents the mean values of the percentage of contrast medium removed from the 5-10mm segment. A significant difference was found in the mean percentage of the contrast medium removed in the three groups from the 5-10mm segment (*P*=0.02) and the ProTaper group showed the highest mean percentage of contrast medium removed. The results of Tukey’s test revealed no significant difference between the Neolix and ProTaper (*P*=0.13) or Neolix and Reciproc (*P*=0.66) in 5-10mm segment; however, a significant difference was noted between the ProTaper and Reciproc groups in the mean values (*P*=0.01)( Table 3).

**Table 2:**
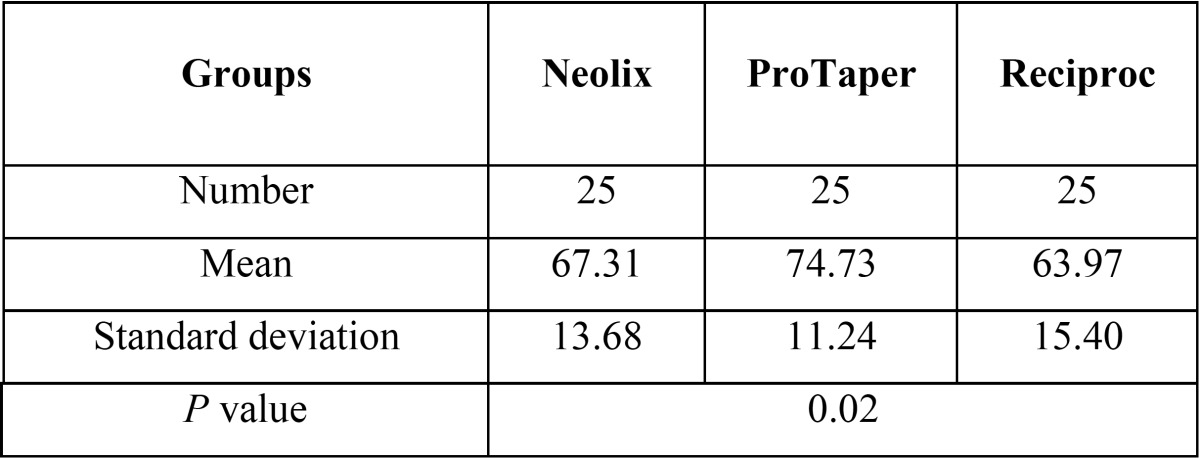
Descriptive statistics of the percentage of medium removed from the root canals in the three groups in 5-10mm segment.

**Table 3:**
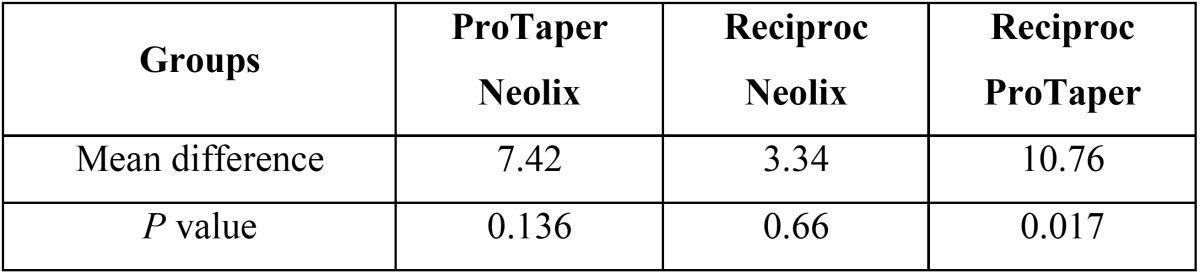
Pairwise comparison of groups for the mean percentage of medium removed from the root canals in 5-10mm segment using Tukey’s test.

The mean percentage of the contrast medium removed from the 0-5mm segment was compared with that in 5-10mm segment in each group by t-test. Based on the results ( Table 4), significant differences were noted in this regard in Neolix (*P*=0.024) and Reciproc (*P*=0.002) groups. In both mentioned groups, the mean percentage of the contrast medium removed from the 0-5mm segment was significantly greater than that in 5-10mm segment; however, this difference was not significant in ProTaper group (*P*=0.069).

**Table 4:**
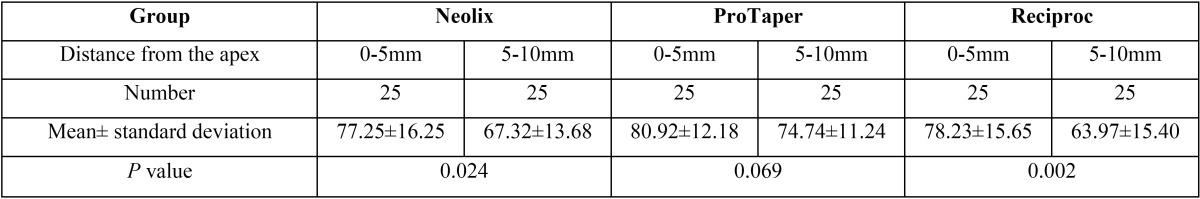
Comparison of the percentage of medium removed from the root canals in 0-5 and 5-10mm segments in the three groups using t-test.

## Discussion

Aside from the complex anatomy of root canals, limitations of preparation techniques are among the main reasons for inefficient cleaning of such root canals (9). In order to assess the efficacy of the newly introduced systems, this study compared the efficacy of ProTaper, which is conventionally used for root canal treatment with that of Reciproc and Neolix single-file rotary systems. The new designs of Neolix and Reciproc files are claimed to have higher flexibility; thus, they should better adapt to root canal walls especially in oval-shaped canals. The final ProTaper file used in our study was F2, which resembles Reciproc and Neolix files in terms of tip diameter and taper since they all have 0.25mm tip diameter and 8% file tip taper.

Different methods have been used for such assessments including scanning electron microscopy (10), reassembly techniques (11), histological sectioning and analysis, micro-computed tomography scan (μCT) (2) and radiography (7). To assess the root canal debridement ability of the three systems, we used the technique described by Ruckman *et al.*, in 2013 (7) for long oval-shaped canals. This method has several advantages such as high accuracy in two-dimensional assessment of root canal debridement efficacy, simplicity, availability and no need for complex equipment. Selection of Vitapex, which is a calcium hydroxide paste containing iodoform, as the radiopaque contrast medium for assessment of root canal debridement was also based on a previous study by Ruckman *et al.* (7). Radiopacity and simple application are among the advantages of Vitapex.

Comparison of the root canal debridement ability of the three systems in 0-5mm segment of long oval-shaped root canals (which is a critical area) revealed no significant difference (*P*=0.3); this finding is in line with that of Ruckman *et al.*, in 2013 (7) but in contrast to the results of Busquim *et al.*, in 2014 (8). Ruckman showed that all three techniques had similar root canal debridement ability in 0-5mm segment, which is in accordance with our results. He attributed the lack of a significant difference in this regard in the apical third to the round cross-section of the apical third in long oval-shaped canals. In addition to this, in our study, similar diameter and taper of the file tips in the three systems might have contributed to this finding. However, Busquim showed that Reciproc single-file system had higher efficacy in the apical third than BioRace multiple-file system, which is in contrast to our result. He attributed this finding to greater taper of Reciproc in apical 3mm compared to the final file of BioRace. The difference between their results and ours is probably due to the use of different multiple-file systems in the two studies (BioRace in their study versus ProTaper in ours).

We noticed a significant difference in 5-10mm segment from the apex in the debridement ability of ProTaper and Reciproc in this segment but ProTaper and Neolix were not significantly different. The difference between Reciproc and Neolix was not significant either. Our findings in this regard were similar to those of Hilaly Eid and Wanees Amin (12), Paque *et al.*, (13), De Deus *et al.*, (14) and Wu *et al.*, (9). They reported that when used in circumferential motion, ProTaper left fewer unprepared areas in oval canals. In our study, the difference between ProTaper and Reciproc may partly be due to the type of motion of these systems since ProTaper shaping files should be used with circumferential brushing motion for better contact to the walls while Reciproc file should be used with reciprocating motion.

Our findings in 5-10mm segment were in contrast to those of Busquim. He attributed his finding to the similar coronal taper of Reciproc file and final file of BioRace whereas in our study the situation was reverse and the diameter and taper of file tips were the same and the files had different coronal tapers.

In our study, no difference was noted in the efficacy of Reciproc and Neolix in 5-10mm segment. The situation was the same for ProTaper and Neolix. Thus, based on our findings, Neolix is probably inferior to ProTaper for preparation of long oval-shaped root canals while has higher efficacy than Reciproc for preparation of these canals; although these differences were not statistically significant. Search of the literature yielded no previous study on Neolix. Neolix is a newly introduced NiTi file with a manufacturing process totally different from that of other NiTi rotary systems known as wire cut electrical discharge machining. The manufacturer claims that this manufacturing process confers high flexibility and surface hardness to the files and combination of these characteristics with rectangular cross-section and cutting edges results in high cutting (shear) efficacy and optimal flexibility enabling the operator to have a suitable tactile sense while performing circumferential filing motion. This is particularly important for cleaning and shaping of oval-shaped root canals. On the other hand, Reciproc system is manufactured by M-Wire NiTi system, which, according to the manufacture, confers increased fatigue resistance and flexibility to the files. Considering all the above, it may be concluded that Neolix, due to higher flexibility, enables better contact with the buccal and lingual walls (most difficult areas in long oval canals to clean) with brushing motions and thus, has superior efficacy to Reciproc in preparation of long oval canals. Moreover, as stated earlier, previous studies (9,13,15) have shown that reciprocating motion, which is the main motion in the Reciproc system, has lower efficacy for preparation of long oval-shaped canals.

In our study, the prepared root canal areas were greater in 0-5mm segment compared to 5-10mm segment, which was similar to the results of Weiger *et al.* (16). The cross-section of oval root canals in the apical third is rounder and smaller, providing better adaptation to the files and resulting in better debridement with rotary instruments.

In general, none of the systems used in our study completely debrided the oval-shaped canals, which is in line with the findings of previous studies (1-3,4,16).

## Conclusions

Neolix single-file system may be a suitable alternative to ProTaper multiple-file system in debridement of long oval shaped canals.
